# Feasibility of Xpert Ebola Assay in Médecins Sans Frontières Ebola Program, Guinea

**DOI:** 10.3201/eid2202.151238

**Published:** 2016-02

**Authors:** Rafael Van den Bergh, Pascale Chaillet, Mamadou Saliou Sow, Mathieu Amand, Charlotte van Vyve, Sylvie Jonckheere, Rosa Crestani, Armand Sprecher, Michel Van Herp, Arlene Chua, Erwan Piriou, Lamine Koivogui, Annick Antierens

**Affiliations:** Médecins Sans Frontières, Brussels, Brussels, Belgium (R. Van den Bergh, P. Chaillet, R. Crestani, A. Sprecher, M. Van Herp, A. Antierens);; Hôpital National Donka, Conakry, Guinea (M.S. Sow);; Comité de Recherche Ebola de Guinée, Conakry (M.S. Sow);; Médecins Sans Frontières, Conakry (M. Amand, C. van Vyve, S. Jonckheere);; Médecins Sans Frontières, Geneva, Switzerland (A. Chua);; Institute of Infectious Diseases and Epidemiology, Singapore (A. Chua);; Médecins Sans Frontières, Amsterdam, Amsterdam, the Netherlands (E. Piriou);; Institut National de Santé Publique, Conakry (L. Koivogui)

**Keywords:** Ebola, Ebola virus disease, Ebola virus, viruses, outbreak, diagnosis, diagnostic techniques, Xpert Ebola Assay, PCR, Médecins Sans Frontières, Guinea

## Abstract

Rapid diagnostic methods are essential in control of Ebola outbreaks and lead to timely isolation of cases and improved epidemiologic surveillance. Diagnosis during Ebola outbreaks in West Africa has relied on PCR performed in laboratories outside this region. Because time between sampling and PCR results can be considerable, we assessed the feasibility and added value of using the Xpert Ebola Assay in an Ebola control program in Guinea. A total of 218 samples were collected during diagnosis, treatment, and convalescence of patients. Median time for obtaining results was reduced from 334 min to 165 min. Twenty-six samples were positive for Ebola virus. Xpert cycle thresholds were consistently lower, and 8 (31%) samples were negative by routine PCR. Several logistic and safety issues were identified. We suggest that implementation of the Xpert Ebola Assay under programmatic conditions is feasible and represents a major advance in diagnosis of Ebola virus disease without apparent loss of assay sensitivity.

## Abstract 2

This assay provides results in less time than routine PCR and is equally sensitive.

As of June 28, 2015, the recent Ebola virus disease (EVD) outbreak in West Africa had claimed >11,000 lives, and 27,443 confirmed, probable, and suspected cases have been reported in Guinea, Liberia, and Sierra Leone (*1*). One of the cornerstones of outbreak control has been rapid diagnosis of suspected cases. Timely confirmation of EVD status can lead to more rapid identification of EVD cases (decreasing potential transmission to contacts, and since the advent of treatment trials, expediting provision of potentially life-saving therapeutics); more immediate initiation of contact tracing; and more accurate epidemiologic surveillance. In addition, a rapidly obtained negative test result would decrease the time a suspected case-patient spends in an Ebola treatment center (ETC) or other Ebola-related health facility, and decrease the likelihood of infection with Ebola virus (EBOV) while waiting for the test result.

Since the start of the recent outbreak, international mobile laboratories were rapidly deployed, mainly near ETCs, to confirm the EVD status of suspected patients and of bodies recovered from the community, and to document the status of survivors. Current diagnostic testing is performed by using PCR of RNA extracted from venous blood samples or swab samples (e.g., oral swab samples for deceased patients). Although conventional PCRs have high specificity and sensitivity, the time between sampling and obtaining results can be considerable, in particular in settings in which a laboratory with PCR capacity is not readily available (*2*). Even in settings in which a laboratory is near an ETC, major delays can occur in obtaining results. A point-of-care instrument capable of diagnosing EVD with high sensitivity and specificity would preclude such delays. Even in environments in which control of an outbreak is (nearly) achieved, health structures are likely to be confronted with suspected cases of EVD for a considerable time. Thus, rapid, point-of-care diagnostics are likely to be invaluable in keeping health structures safe.

A novel Ebola diagnostic assay, the Xpert Ebola Assay (Cepheid Inc., Sunnyvale, CA, USA), was recently developed. This assay can be used with the Cepheid GeneXpert System, which is widely used for rapid detection of tuberculosis and rifampin resistance in decentralized settings (*3*). The Xpert Ebola Assay has been approved for emergency use by the US Food and Drug Administration for testing of venous blood samples on the basis of laboratory studies that used venous blood spiked with EBOV (*4*). The World Health Organization has issued a prequalification of the Xpert Ebola Assay (*5*).

However, the feasibility of implementing this technology inside a functioning ETC (compared with an external laboratory), and the added value to Ebola programs are not known. Therefore, we conducted a study that assessed the added value of using the Xpert Ebola Assay in a Médecins Sans Frontières (MSF) ETC during the recent EVD outbreak in West Africa. Specifically, we compared total test time and time for obtaining results for the Xpert Ebola Assay with those for a routine in-house Ebola PCR used at the ETC to document any discordant results between the 2 tests and document information regarding biosafety and logistic and human resource requirements for implementation of the Xpert Ebola Assay.

## Methods

### Study Design

We conducted a cross-sectional study of laboratory analysis of paired venous blood samples. This study included a limited user satisfaction survey conducted by using semistructured interviews.

### Ethics Statement

Verbal informed consent was obtained for all study participants, including patients who provided blood samples for analysis and laboratory staff who participated in user evaluation of the Xpert Ebola Assay. The study was approved by the Ethics Review Board of MSF (Geneva, Switzerland) and the Comité de Recherche Ebola and Comité National d’Ethique pour le Recherche en Santé (Conakry, Guinea).

### Setting

The study was conducted in the MSF-managed Donka ETC in Conakry, Guinea. The Donka ETC was opened in March 2014 at the start of the outbreak in West Africa. It has 30 beds (85 at its peak capacity during October 2014–January 2015). By week 28 of 2015, Donka had admitted 818 patients with confirmed cases of EVD and discharged 789 patients (372 died and 417 recovered). MSF ETCs are based on the principle of providing patient care and support and isolating EVD-positive patients to break the chain of transmission. The centers aim to provide controlled access to patients or contaminated areas; controlled movement of staff, patients, and visitors inside these areas; disinfection facilities for persons leaving contaminated areas; and safe disposal of contaminated waste.

During the study, the Donka ETC hosted a clinical trial of treatment for EVD by transfusion of convalescence plasma. This trial (Ebola_Tx) was conducted by the Institute for Tropical Medicine (Antwerp, Belgium) (*6*). For routine diagnostics, the Donka ETC had samples tested at the Laboratoire National des Fièvres Hémorrhagiques, Gamal Abdel Nasser University of Conakry (Conakry, Guinea), which is supported by the Institut Pasteur (Dakar, Senegal). This laboratory was located in the same compound as the Donka ETC, but was not part of the center. An in-house, real-time PCR specific for the nucleocapsid protein gene of EBOV was used for routine diagnostics (*7*).

### Sample Processing

Blood samples were obtained at the same time as the convalescent-phase plasma trial at the Donka ETC was conducted. Persons who fulfilled the definition for having suspected EVD were counselled by a health promotion team and then provided blood obtained by venipuncture. Venous blood for routine diagnosis was collected in a 4-mL serum tube (Vacutainer; Becton Dickinson, Franklin Lakes, NJ, USA). An additional 2-mL blood sample was obtained from consenting persons into an EDTA tube (Vacutainer). Persons who did not provide consent, children <2 years of age, and persons who were not able to provide an additional blood sample (e.g., severely dehydrated persons) were excluded from the study (number was not recorded). For consenting persons admitted to the ETC after a positive routine diagnosis was made, additional 2-mL ml blood samples were collected into EDTA tubes 24 or 48 hours after a transfusion with convalescent-phase plasma and during convalescence (after 3 days without symptoms), each time in parallel with the 4-mL blood sample obtained for routine PCR testing.

Serum tubes were directly transferred to the routine laboratory for processing and testing. Samples in EDTA tubes were processed in a specifically developed MSF laboratory in the ETC that contained separated high-risk and low-risk areas. Samples in the high-risk area (deposited by ETC staff using complete personal protective equipment after sampling) could be handled by a technician in the low-risk area if the technician used sealed gloves and worked through a partition (glovebox-type setup). Technicians who worked only in the low-risk area did not require full personal protective equipment, used only latex gloves, and were dressed in scrubs and plastic boots in the laboratory (e.g., when using the Xpert Ebola Assay or handling inactivated samples). When these technicians worked through the partition and used the glovebox, they also wore surgical gowns and an additional layer of surgical gloves. The laboratory was staffed by 2 or 3 technicians at all times during the study.

In the high-risk area, 100-μL samples from each EDTA tube were directly transferred by using an automatic pipette with filter tips into a Cepheid sample reagent vial containing 2.5 mL of inactivating agent (4.5 mol/L guanidine thiocyanate). After 20 min of inactivation (twice the 10 min recommended by the manufacturer), the vial was transferred to the low-risk area in accordance with all biosafety procedures (decontamination of the vial in the high-risk area in a 0.5% hypochlorite solution during inactivation, and by incubation for 20 min in a 0.5% hypochlorite solution in the low-risk area, as standard procedure for all material transferred from the high-risk to the low-risk zone; total time of 40 min). Subsequently, 1 mL of inactivated sample was transferred from the vial into the Xpert Ebola Assay cartridge. The cartridge was then inserted into the Cepheid GeneXpert instrument, and testing was conducted according to the manufacturer’s recommendations.

### GeneXpert Setup

The Cepheid GeneXpert setup has been in use in Guinea for tuberculosis diagnosis for several years. It consists of a GeneXpert instrument, personal computer, and disposable fluidic cartridges. Each instrument contains 4 individually accessible modules that are capable of independently performing testing. A specific cartridge (Xpert Ebola) specific for the EBOV Zaire strain was developed to target highly conserved sequences in the nucleocapsid protein (NP) and glycoprotein (GP) genes (*5*). The Xpert Ebola assay is fully automated and cartridge based (closed system) and includes automated controls for interference with the PCR and adequacy of sample input. The only manual step is inactivation of the blood sample and transfer to the Cepheid cartridge; sample processing (RNA extraction), reverse transcription, real-time PCR amplification, and detection of TaqMan probes are then performed automatically. Results are expressed as positive or negative, and a cycle threshold (C_t_) for both gene targets was also calculated by the software automatically. The results for the NP gene were taken as the final GeneXpert result; no interpretation of amplification characteristics was conducted.

### Data Collection and Analysis

All data was entered into a dedicated electronic data registry (Excel; Microsoft, Redmond, WA, USA), and data validation was performed by random checking of >10% of all records. Information was collected regarding patient characteristics and timing of each step in the Xpert Ebola Assay. For samples used as comparators, only time of sample submission to the laboratory and time of result could be recorded. Analysis was conducted by using EpiData Analysis version 2.2.2.183 software (EpiData Association, Odense, Denmark). Total times were calculated for each step of the testing procedure, descriptive data was presented as summary statistics, and differences in median timings were assessed by using the paired-sample Wilcoxon signed-rank test. Levels of significance were set at p<0.05.

Additional data for user experience was collected by using a structured questionnaire that included several statements about practical use of the Xpert Ebola Assay. Agreement was assessed on a scale of 1 through 5 (1 = complete disagreement to 5 = complete agreement). This questionnaire was completed twice by all 5 laboratory technicians who used the Xpert Ebola Assay: once at the start of work, and once after several weeks of work.

## Results

### Patient and Sample Characteristics

**Figure 1:**
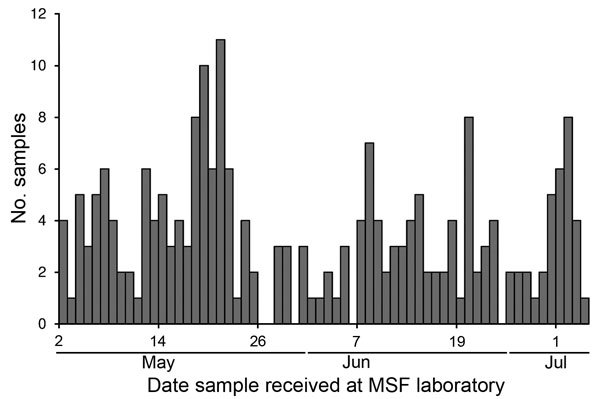
Frequency of sampling for Ebola virus at Médecins Sans Frontières (MSF) Donka Ebola Treatment Center, Conakry, Guinea, May–June 2015.

Data were analyzed for all samples collected during May 2−July 4, 2015; a total of 218 samples were collected from 148 persons (Figure 1). All samples collected were venous blood samples. Characteristics of samples and patients from which they were recovered are shown in Table 1. Median delay between estimated time of onset of symptoms and admission to the ETC was 3 days (interquartile range [IQR] 1–6 days).

**Table 1:** Characteristics of 148 patients and 218 blood samples collected for analysis by Xpert Ebola Assay at Médecins Sans Frontières Donka Ebola Treatment Center, Conakry, Guinea, May–June 2015

Characteristic	No. (%)
Patient sex	
F	59 (40)
M	89 (60)
Patient age, y	
2–4	15 (10)
5–18	19 (13)
19–45	84 (57)
46–64	16 (11)
>65	11 (7)
Not recorded	3 (2)
Sample type	
Diagnosis 1*	147 (67)
Diagnosis 2 (confirmation)	52 (24)
After transfusion	12 (6)
Convalescent phase	7 (3)

*One patient (2-year-old boy) did not have an initial diagnostic sample obtained for the study.

### Timeliness of Testing

**Figure 2:**
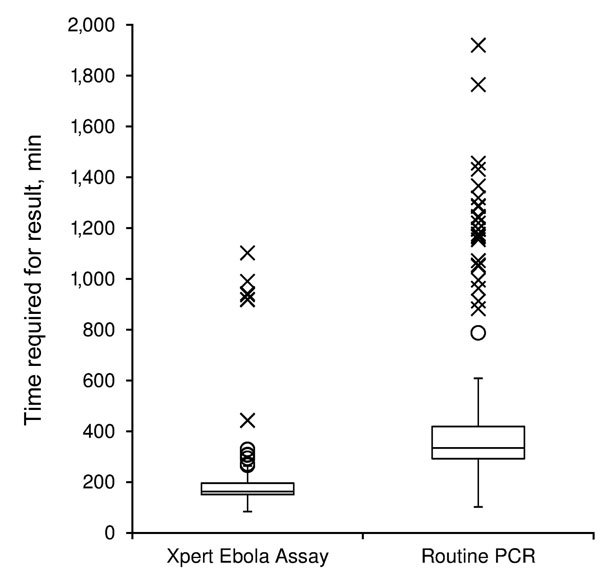
Tukey boxplot of time required from receiving sample in laboratory to obtaining results by Xpert Ebola assay and routine PCR at Médecins Sans Frontières Ebola Donka Treatment Center, Conakry, Guinea, May–June 2015. Boxes indicate first and third quartiles; vertical dashed lines indicate medians; whiskers indicate 1.5 times interquartile ranges (IQRs); asterisks indicate outliers >3 times the IQR; and circles indicate outliers 1.5–3 times the IQR.

Median time of each step in the testing process is show in Table 2. The median run time of the instrument was 94 min (IQR 94–95 min) for all successful runs (n = 218). Ten (5%) tests had to be repeated because of technical issues (8 power failures that were not buffered by an uninterrupted power supply, 1 temperature-related failure, and 1 invalid result); all repeated tests were successful. A full comparison of each step of the testing process could not be performed because data were only available from the routine laboratory on reception of the sample and provision of the results to the clinical staff of the ETC. However, when we compared the total result notification time (time between reception of sample in the MSF laboratory and availability of results), the median time for results to be available was reduced from 334 min (IQR 293–419 min) in the routine laboratory that used the in-house PCR to 163 min (IQR 151–196 min) in the laboratory that used the Xpert Ebola Assay (p<0.0001) (Figure 2). These times included the time required for repeat Xpert Ebola Assay for the 10 failed runs (n = 228 for the Xpert Ebola Assay); 11 samples were excluded from routine laboratory data because the time for obtaining a result was not recorded (n = 207 for in-house PCR).

**Table 2:** Timing of steps in Xpert Ebola Assay for blood samples collected at Médecins Sans Frontières Donka Ebola Treatment Center, Conakry, Guinea, May–June 2015*

Step	Median (IQR), min
Sampling obtained to sample received at laboratory†	11 (5–20)
Sample received to inactivation	14 (8–28)
Inactivation to start of assay	49 (43–56)
Start of assay to end of assay	94 (94–95)
End of assay to result available	2 (1–8)

*IQR, interquartile range. †Only 67 samples were included; time of collection was not recorded for other samples.

### Test Discordance

Of 218 samples tested, the Xpert Ebola Assay identified 26 (12%) positive samples: 8 (5%) of 147 at initial diagnosis, 12 (100%) of 12 after transfusion, and 6 (86%) of 7 at convalescence. The routine laboratory identified 18 (69%) of the 26 positive samples identified by the Xpert Ebola Assay. No discordance was observed for diagnostic samples (Table 3), and no samples identified as negative in the Xpert Ebola Assay were identified as positive by the routine laboratory. The 8 samples identified as positive in the Xpert Ebola Assay and as negative by the routine laboratory (5 samples obtained during convalescence and 3 samples obtained after transfusion) had low viral loads (range C_t_ 33.0–40.8 for the NP gene) and were obtained from 4 patients. Detailed results for each of these patients are provided in the Technical Appendix.

**Table 3:** Blood samples identified as positive for Ebola virus by Xpert Ebola Assay at Médecins Sans Frontières Donka Ebola Treatment Center, Conakry, Guinea, May–June 2015

Sample type	No. positive by Xpert Ebola Assay	No. (%) positive by routine PCR
Total	26	18 (69)
Diagnosis 1	8	8 (100)
Diagnosis 2	0	0
After transfusion	12	9 (75)
Convalescent phase	6	1 (17)

**Figure 3:**
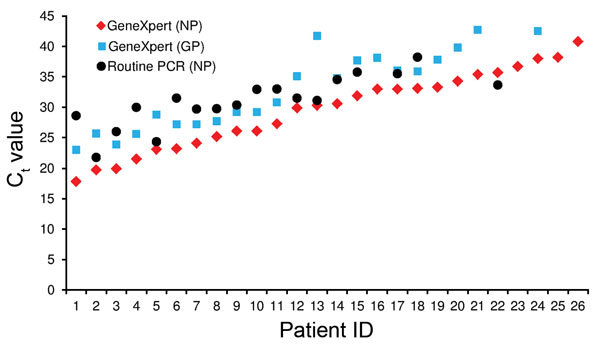
Ebola virus cycle threshold (C_t_) values for GeneXpert Ebola Assay (nucleocapsid protein [NP] and glycoprotein [GP] genes) and routine PCR (NP gene) for patient samples identified as positive for Ebola virus by Xpert Ebola Assay at Médecins Sans Frontières Donka Ebola Treatment Center, Conakry, Guinea, May–June 2015. ID, identification.

The C_t_ for the NP gene in the Xpert Ebola Assay was compared with that for the GP gene and that for the NP gene in routine PCR (Figure 3). Although a clear correlation was observed (Pearson *r* = 0.95 with the GP gene and *r* = 0.80 with the NP gene in routine PCR), with only 1 exception, the C_t_ was lower (a higher viral load detected) in the Xpert Ebola Assay for the NP gene (Figure 3). Of 18 samples that had positive results for both tests, 12 (67%) had a difference of >3 C_t_ values, or approximately a 10-fold difference. When results were transformed to a linear scale, we found that viral load assessed by using the Xpert Ebola Assay (NP) was a median 22-fold higher (IQR 5–64 fold) than viral load assessed by the routine PCR (NP).

### User Satisfaction and Lessons Learned

**Figure 4:**
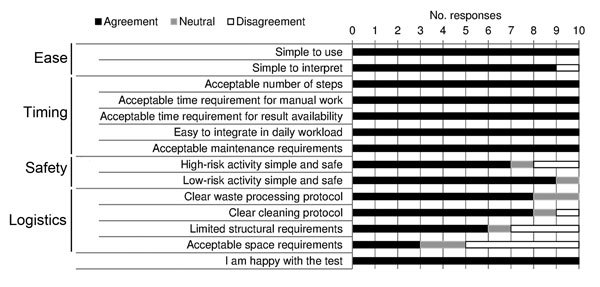
Laboratory staff feedback on key aspects of implementation of Xpert Ebola assay at Médecins Sans Frontières Donka Ebola Treatment Center, Conakry, Guinea, May–June 2015.

Five laboratory technicians used the Xpert Ebola Assay during the study. One of these technicians had experience with the assay for diagnosis of tuberculosis. Opinions were queried on several aspects of the practical implementation of the assay (Figure 4). An overall high appreciation of ease of use was observed, and concerns regarding logistical aspects (storage space, cleaning) and safety aspects (manipulation of inactivated samples in the low-risk zone) were observed (Table 4).

**Table 4:** Main user concerns for Xpert Ebola Assay at Médecins Sans Frontières Donka Ebola Treatment Center, Conakry, Guinea, May–June 2015

Concern
Biosafety
• There were difficulties in preventing the rim of the inactivation vial from being touched by the tip of the pipette (which was contaminated with blood). Because material on the rim is not inactivated, this situation could be a considerable biohazard; the only strategy to avoid this situation was close observation of the vial rim by laboratory staff.
• Three incidents were reported in which vials containing inactivation fluid were dropped because of difficulties in handling vials with required biosecurity gloves; no incidents occurred with sample already added.
• The decontamination process for vials and transferring these vials to a low-risk zone did not compromise assay performance.
Instruments
• It was not possible to automatically export Xpert Ebola Assay data into an existing database; thus, manual coding was required.
• More detailed information on assay characteristics (e.g., PCR efficiency) was not available for users.
• Compatibility issues were identified when we attempted to set up a laboratory in which French was spoken; this problem remained unresolved throughout the study.
Logistics
• Power failures could usually be corrected by an uninterrupted power supply, which could support a full Xpert Ebola Assay run without depleting >25% of the power supply. However, the computer to which the Xpert Ebola Assay was linked could not be supported by the uninterrupted power supply and ran on its own battery. On several occasions, the uninterrupted power supply was reconfigured because of daily switching between day and night generators and failed to support the assay.
• Failure of air conditioning resulted in ambient temperatures of 28°C–31°C, but this failure did not have a major effect on instruments used (manufacturer’s recommendations are to work at a temperature <30°C); only 1 run failure was observed.
• At the time of the study, insufficient information was available on storage conditions for reagents.
• Sufficient space was needed to store all equipment and consumable materials and handle chemicals (e.g., chlorine solution) for sample processing. However, the field laboratory (area ≈2.5 m × 5.5 m) was too small for storage of all materials.

## Discussion

This study represents an analysis of implementation of the Xpert Ebola Assay in field conditions and assesses the practical aspects of its implementation and the added value in a functional EVD program in West Africa. Our results demonstrate the feasibility of introducing the Xpert Ebola Assay as a routine diagnostic tool for venous blood samples in an ETC and indicate a major decrease in time needed for obtaining results by using this point-of-care device. This assay halves the time required for the in-house PCR test used by the routine laboratory in the study setting without an apparent loss in sensitivity for detection of EVD cases. Sample sizes of positive cases were limited, but this assay seemed capable of better identifying positive cases with a low viral load than the routine diagnostic PCR used in the national laboratory in Donka. Thus, this assay might have the potential for earlier detection of EVD cases, although in this analysis, differences were only observed for convalescent-phase cases.

The longer time required for detection of convalescent-phase cases might have repercussions for patient discharge and length of stay in the ETC. More wide-scaled studies of Xpert Ebola Assay sensitivity and specificity in patient samples compared with in vitro−spiked samples used for validation are recommended, in particular, for diagnostic samples. In addition, given the higher C_t_ for the GP gene than for the NP gene, an in-depth analysis of associations between Ct values in different systems for different targets and viral loads might be indicated.

Several concerns were raised on the practical implementation of this novel system, in particular concerning biosafety and logistics, which might be relevant to persons seeking to implement a similar system for EVD diagnostics. At the level of sample collection, the approach does not differ from sampling for routine PCR diagnosis, and all standard ETC precautions (personal protective equipment, safe handling, safe packaging of samples) need to be in place as a prerequisite for implementing the Xpert Ebola Assay. In addition, laboratory technicians need comprehensive training on the safe manipulation of the instrument and samples if they do not have experience working with EBOV biologic material. In terms of logistics, implementers need to address the need for sufficient working space, provision of air-conditioning, a robust system for managing power failures, and a safe way of handling the critical step of transferring the biologic material to the inactivation vial.

Other studies have reported similar advances in providing point-of-care diagnostics in Ebola programs, focusing mainly on rapid diagnostic tests (*8**,**9*). Although such technology is expedient as a screening tool, approaches that rely on antigen detection might have difficulties matching the sensitivity of molecular techniques. In an outbreak setting, even 1 false-negative result, which would result in not identifying an EVD case, could initiate a new chain of transmission. Thus, the Xpert Ebola Assay might represent a promising strategy toward rapid EVD diagnosis because its sensitivity is at least equivalent with that of conventional PCR. In settings in which an outbreak is being brought under control, this assay might be of considerable value because it has been shown to be implementable at the primary care level for treatment of tuberculosis (*10**,**11*).

The Xpert Ebola Assay could represent an advantageous strategy for screening in health structures that have no cases of EVD if (and only if) the following conditions can be met: 1) safe handling of samples of patients with suspected cases of EVD (including full personal protective equipment); 2) capacity to identify suspected case-patients and refer confirmed case-patients for treatment; and 3) capacity to practically manage the assay at the level of laboratory biosafety, logistics, and training of laboratory staff. However, such conditions may not easily be met under operational conditions. Validation of the Xpert Ebola Assay for finger-prick blood and swab samples, both at the level of test performance and of biosafety and feasibility, might facilitate such decentralized use of the test.

The study had some limitations. A national laboratory providing routine diagnosis was used as a comparative setting. However, this laboratory used an in-house PCR for diagnostic purposes. Thus, sample turnaround time might have not been representative for other laboratories deployed for EVD diagnostics. A more detailed breakdown of the timing of each step in the routine procedure would have enabled a more refined comparison and identification of potential room for improvement, but this information was not available. However, by reporting the breakdown of time intervals for the Xpert Ebola Assay, we hoped to provide a reference for other potential implementers.

In addition, because the study was conducted during May−June 2015, caseloads were relatively low at the Donka ETC and were not comparable to overwhelming caseloads observed in the EVD outbreak in West Africa during August−September 2014. Nevertheless, the staff feedback on time management was unequivocally positive, and it seems plausible that the time gains observed in this study would also be apparent under higher caseloads. However, a GeneXpert instrument with a higher number of modules could be required in settings with high caseloads. If the available 16-module instrument was used and >4 runs/day were conducted, >64 samples/day could be processed.

In conclusion, we show that implementation of the Xpert Ebola Assay under programmatic conditions in an MSF ETC in Guinea is feasible and represents a major decrease in time required to obtain results and a possible increase in sensitivity compared with routine diagnostic procedures that use PCR in this setting. Wider implementation of the Xpert Ebola Assay is recommended for facilities capable of supporting safe sampling and patient management if training of laboratory staff and standard operating procedures can be provided. Additional research into sensitivity and specificity of this test for patient samples is encouraged.

**Technical Appendix.** Ebola virus cycle threshold (C_t_) for Xpert Ebola Assay (nucleocapsid protein [NP] and glycoprotein [GP] genes) and for routine PCR (NP gene) for samples identified as positive for Ebola virus from 8 patients by Xpert Ebola Assay at Médecins Sans Frontières Donka Ebola Treatment Center, Conakry, Guinea, May–June 2015.
