# Supplementary material for: Feasibility of Xpert Ebola Assay in Médecins Sans Frontières Ebola Program, Guinea
Source: Emerg Infect Dis. 2016 Feb;22(2):210–6. doi: 10.3201/eid2202.151238 (PMC4734513; doi:10.3201/eid2202.151238)
Supplement: Supplementary file 1 — Technical Appendix. Ebola virus cycle threshold (Ct) for Xpert Ebola Assay (nucleocapsid protein [NP] and glycoprotein [GP] genes) and for routine PCR (NP gene) for samples identified as positive for Ebola virus from 8 patients by Xpert Ebola Assay at Médecins Sans Frontières Donka Ebola Treatment Center, Conakry, Guinea, May–June 2015. [file 15-1238-Techapp-s1.pdf]

Article DOI: http://dx.doi.org/10.2301/eid2202.151238

Feasibility of Xpert Ebola Assay in Médecins Sans Frontières Ebola Program, Guinea

Technical Appendix

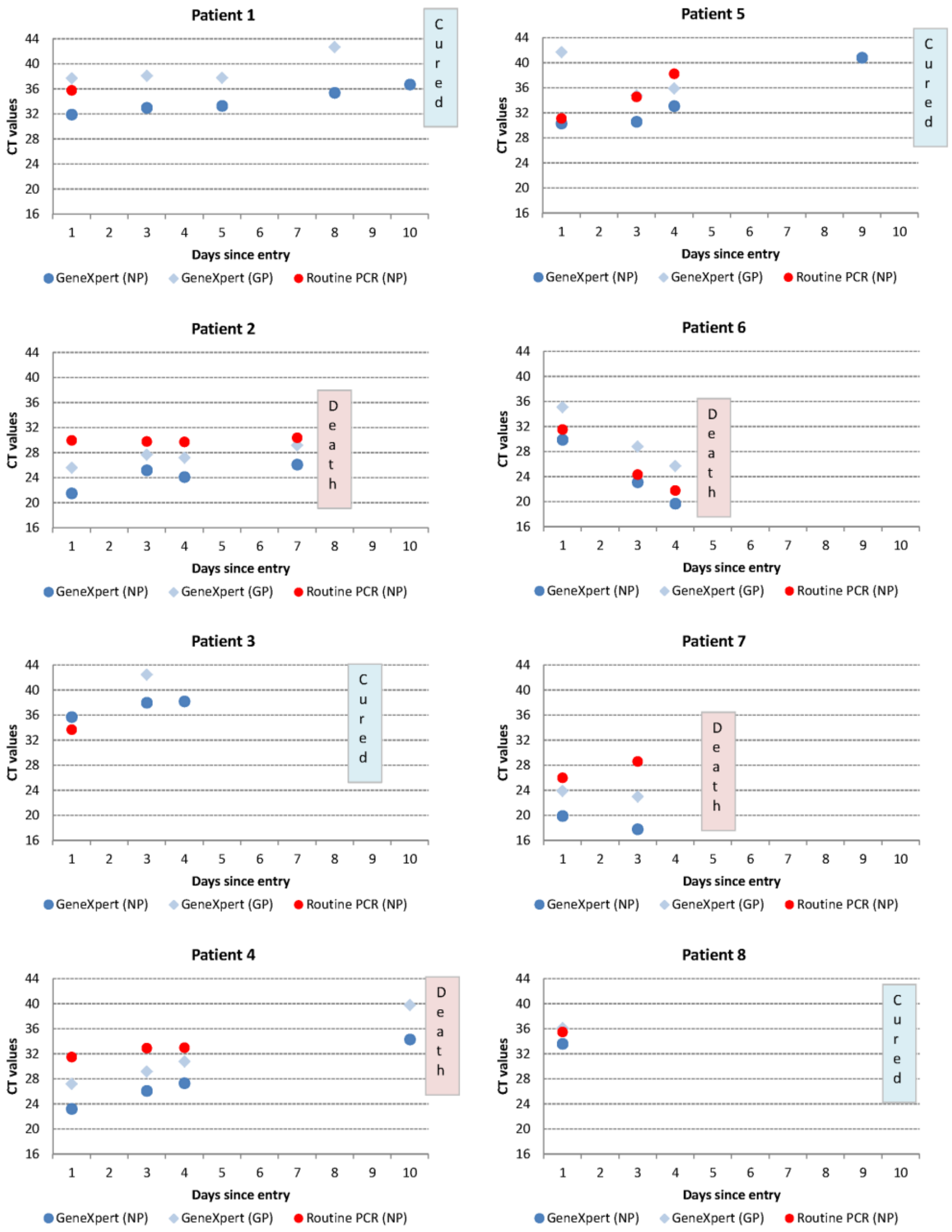

Technical Appendix Figure. Ebola virus cycle threshold (C_t) for Xpert Ebola Assay (nucleocapsid protein [NP] and glycoprotein [GP] genes) and for routine PCR (NP gene) for samples identified as positive for Ebola virus from 8 patients by Xpert Ebola Assay at Médecins Sans Frontières Donka Ebola Treatment Center, Conakry, Guinea, May–June 2015.
